# From seropositivity to neurosyphilis: clinical spectrum and diagnostic classification in hospitalized neurology patients: a 16-year retrospective study

**DOI:** 10.3389/fneur.2026.1911231

**Published:** 2026-08-25

**Authors:** Duygu Özkan Yaşargün

**Affiliations:** Department of Neurology, University of Health Sciences, Haydarpaşa Numune Training and Research Hospital, İstanbul, Türkiye

**Keywords:** cerebrospinal fluid VDRL, confirmed neurosyphilis, magnetic resonance imaging, neurosyphilis, probable neurosyphilis, syphilis seropositivity, *Treponema pallidum*

## Abstract

**Background:**

Neurosyphilis remains a diagnostic challenge because of its heterogeneous neurological manifestations and the absence of a universally accepted diagnostic gold standard. Diagnostic categories such as confirmed and probable neurosyphilis may help stratify syphilis-seropositive patients according to clinical and cerebrospinal fluid findings. This study aimed to evaluate syphilis seropositivity among hospitalized neurology patients and to compare clinical, radiological, and discharge neurological characteristics across diagnostic groups.

**Methods:**

This single-center retrospective observational study included adult patients hospitalized in the neurology ward between January 2010 and January 2026 who were identified as having positive serum treponemal serology. Patients were classified as confirmed neurosyphilis, probable neurosyphilis, or non-neurosyphilis seropositivity.

**Results:**

Among 9,172 hospitalized neurology patients who underwent serum TPHA testing during the study period, 25 eligible patients with syphilis seropositivity were included in the analysis. Ten patients were classified as confirmed neurosyphilis, five as probable neurosyphilis, and ten as non-neurosyphilis seropositivity. Age, sex distribution, vascular ischemia on admission, HIV status, and CT angiography vascular involvement did not differ significantly among the groups. MRI findings suggestive of neurosyphilitic involvement and positive neurological examination at discharge differed significantly among the diagnostic groups (both *p* = 0.001). Pairwise comparisons showed that these differences were significant only between the confirmed neurosyphilis and non-neurosyphilis seropositivity groups.

**Conclusion:**

Although syphilis seropositivity was uncommon among hospitalized neurology patients, a substantial proportion (60%) of seropositive individuals fulfilled the predefined criteria for confirmed or probable neurosyphilis. Our findings demonstrate that syphilis seropositivity should not be considered synonymous with neurosyphilis and highlight the importance of a comprehensive diagnostic evaluation integrating clinical presentation, cerebrospinal fluid findings, and neuroimaging. Syphilis testing remains a crucial component in the diagnostic evaluation of unexplained neurological syndromes in contemporary practice.

## 1 Introduction

Syphilis is a systemic infection caused by *Treponema pallidum* and remains clinically relevant because of its recent resurgence and broad spectrum of manifestations (1). Although the widespread use of penicillin markedly reduced its burden during the twentieth century, contemporary data indicate that syphilis has re-emerged in several regions and populations (1, 2). As a systemic infection, syphilis may also involve organs outside the nervous system. Renal involvement is uncommon but may present with nephritis, membranous nephropathy, nephrotic syndrome, and peripheral edema, particularly during secondary syphilis (1, 3, 4). Neurological involvement may occur at any stage of infection and can be either asymptomatic or symptomatic, affecting the meninges, vasculature, cranial nerves, spinal cord, or brain parenchyma (1, 5, 6). Because of this heterogeneity, neurosyphilis continues to be regarded as a “great imitator” in neurological practice (6, 7).

The diagnosis of neurosyphilis remains challenging. There is no universally accepted gold standard, and diagnostic assessment usually relies on the integration of neurological findings, serum treponemal serology, and cerebrospinal fluid (CSF) abnormalities (6, 8). Although a reactive CSF-VDRL is highly specific and supports a confirmed diagnosis, its limited sensitivity means that a negative result does not exclude neurosyphilis in patients with compatible neurological manifestations and inflammatory CSF findings (2, 6–9). Therefore, diagnostic categories such as confirmed, probable, presumptive, or likely neurosyphilis have been used in clinical studies and diagnostic frameworks to reflect varying levels of diagnostic certainty (6, 7, 9–11).

Previous studies have described the clinical spectrum of neurosyphilis, including asymptomatic, ocular, psychiatric, cognitive, meningovascular, and parenchymal presentations (1, 6, 11–13). Although some studies have compared symptomatic neurosyphilis with syphilis/non-neurosyphilis groups, data specifically evaluating confirmed, probable, and non-neurosyphilis seropositivity categories in hospitalized neurology patients remain limited (9). This study aimed to evaluate the frequency of syphilis seropositivity among hospitalized neurology patients and to compare demographic, clinical, radiological, and discharge neurological characteristics among patients classified as confirmed neurosyphilis, probable neurosyphilis, and non-neurosyphilis seropositivity.

## 2 Methods

### 2.1 Study design and setting

This was a single-center, retrospective, observational study. The study included adult patients who were hospitalized between January 2010 and January 2026 and were identified as having positive serum treponemal serology during their inpatient neurological evaluation. This study was conducted in accordance with the principles of the Declaration of Helsinki and was approved by the Ethics Committee for Non-interventional Clinical Studies of Haydarpasa Numune Research and Training Hospital, Istanbul, Turkey (Date: April 28, 2026; Approval No. HNEAH-KAEK 2026/99).

### 2.2 Study population

**Figure 1:**
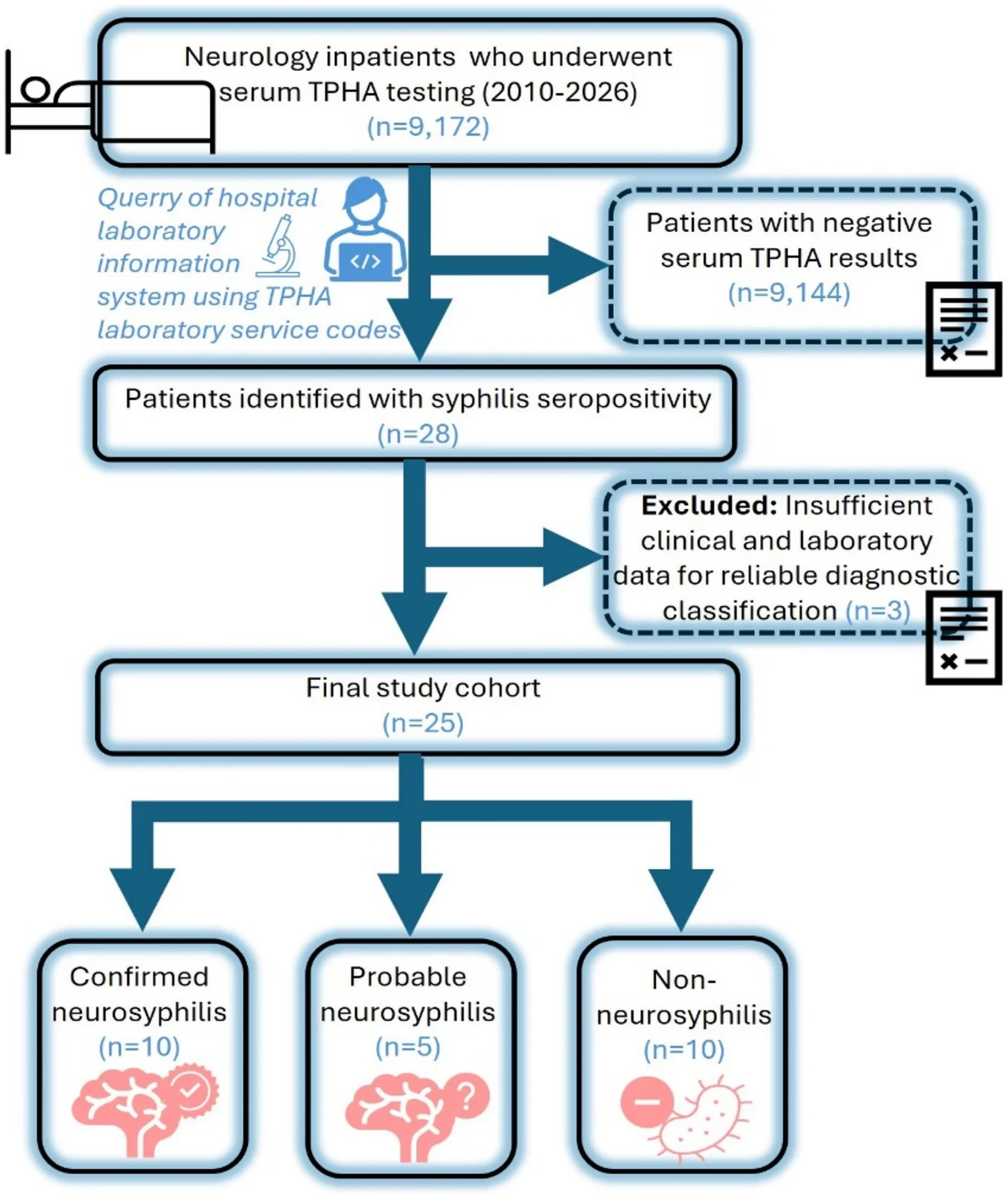
Flowchart of patient selection and classification into the diagnostic groups.

During the study period (January 2010 to January 2026), adult patients (≥18 years) hospitalized in the neurology ward were retrospectively evaluated. In routine clinical practice, serum syphilis serology was not performed systematically in all hospitalized patients but was requested at the discretion of the treating neurologist in patients with unexplained or atypical neurological presentations, including ischemic stroke, intracerebral hemorrhage, myelopathy, headache, dizziness, cognitive or behavioral changes, and other neurological syndromes in whom syphilis was considered in the differential diagnosis. At our institution, the *Treponema pallidum* hemagglutination assay (TPHA) is the routine treponemal screening test, whereas fluorescent treponemal antibody absorption (FTA-ABS) testing is not available. During the study period, serum TPHA testing was performed in 9,172 hospitalized neurology patients based on clinical indication. To identify all eligible cases, the hospital laboratory information system was queried using the relevant TPHA laboratory service codes. A total of 28 patients with positive serum TPHA were identified through this electronic search. Three patients were excluded because the available clinical and laboratory data were insufficient to permit reliable diagnostic classification. The remaining 25 patients constituted the final study cohort. The complete electronic medical records of the included patients were reviewed individually, including admission notes, discharge summaries, laboratory findings, neuroimaging, cerebrospinal fluid findings, treatment records, and clinical outcomes. Based on clinical presentation, serum and cerebrospinal fluid findings, neuroimaging, patients were classified into one of three diagnostic groups: confirmed neurosyphilis, probable neurosyphilis, or non-neurosyphilis seropositivity. The overall patient selection and diagnostic classification process is illustrated in Figure 1.

### 2.3 Data collection and variables

Data were retrospectively extracted from hospital records, laboratory records, and neuroimaging archives. The following variables were included in the final analysis: age, sex, vascular ischemic presentation on admission, HIV status, magnetic resonance imaging findings suggestive of neurosyphilitic involvement, CT angiography findings suggestive of vascular involvement, neurological examination status at discharge, serum VDRL titer category, and final diagnostic group. CSF VDRL testing was performed in the hospital microbiology laboratory. Test results were reported qualitatively (reactive/non-reactive) rather than as quantitative titers. Reactive CSF VDRL results were accepted as positive laboratory evidence of confirmed neurosyphilis and interpreted together with the patient’s clinical presentation and other CSF findings. Serum VDRL titers were available for 22 patients and were recorded as exact dilution values. For exploratory comparisons, titers were dichotomized as <1:16 and ≥1:16. This threshold was selected to distinguish lower from relatively higher nontreponemal antibody titers while maintaining analyzable group sizes in the small cohort and was not regarded as a diagnostic criterion for neurosyphilis.

### 2.4 Diagnostic classification

Patients were categorized into three groups based on predefined clinical, serological, and cerebrospinal fluid criteria: confirmed neurosyphilis, probable neurosyphilis, and non-neurosyphilis seropositivity. The first two categories were based on contemporary guideline recommendations, whereas the non-neurosyphilis seropositivity group was introduced as a study-specific descriptive category to include seropositive patients who did not meet diagnostic criteria for neurosyphilis.

Confirmed neurosyphilis was defined as the presence of neurological symptoms, positive serum treponemal testing, and positive cerebrospinal fluid VDRL.

Probable neurosyphilis was defined by neurological manifestations considered clinically compatible with neurosyphilis, reactive serum treponemal serology, a nonreactive CSF-VDRL test, and CSF pleocytosis (>5 cells/μL) and/or elevated CSF protein (>0.50 g/L) (14). Mild isolated CSF abnormalities were not considered sufficient for classification as probable neurosyphilis in the absence of a clinically compatible neurological presentation.

Non-neurosyphilis seropositivity was defined as reactive serum treponemal serology in patients who did not fulfill the predefined criteria for confirmed or probable neurosyphilis. This group was included as a study-specific descriptive category and was not intended to represent a distinct diagnostic entity.

The diagnostic criteria used for patient classification are summarized in Table 1.

**Table 1:** Diagnostic criteria used for patient classification.

Diagnostic group	Neurological signs or symptoms	Positive serum treponemal test (TPHA)	Positive CSF-VDRL	CSF pleocytosis and/or elevated protein
Confirmed neurosyphilis	✓	✓	✓	Not required
Probable neurosyphilis	✓	✓	✗	✓
Non-neurosyphilis seropositivity	Absent or not compatible	✓	✗	✗

TPHA, Treponema Pallidum Hemagglutination Assay; CSF-VDRL, cerebrospinal fluid Venereal Disease Research Laboratory test. For probable neurosyphilis, CSF pleocytosis and/or elevated protein was required. Imaging findings were not used as formal classification criteria.

### 2.5 Outcomes

The objective was to compare demographic, clinical, radiological, and discharge neurological characteristics among the three diagnostic groups: confirmed neurosyphilis, probable neurosyphilis, and non-neurosyphilis seropositivity. The variables compared among the groups were age, sex, vascular ischemic presentation on admission, HIV status, serum VDRL titer category, MRI involvement suggestive of neurosyphilis, CTA vascular involvement, and positive neurological examination at discharge.

A positive neurological examination at discharge was defined as the presence of any persistent objective neurological abnormality documented at the time of hospital discharge. Patients without a documented residual neurological abnormality were considered to have a negative neurological examination at discharge.

### 2.6 Statistical analysis

Statistical analyses were performed using SPSS software version 21.0. Descriptive statistics were used to summarize the study cohort. Continuous variables were presented as mean ± standard deviation, and categorical variables were presented as numbers and percentages.

The distribution of continuous variables was assessed using the Shapiro–Wilk test and visual inspection of distribution plots. Comparisons among the three diagnostic groups were performed using one-way analysis of variance for normally distributed continuous variables and the Kruskal–Wallis test when appropriate. Categorical variables were compared using the chi-square test or Fisher’s exact test, as appropriate.

For variables showing a statistically significant overall difference among the three groups, pairwise comparisons were performed using Fisher’s exact test with Bonferroni correction. Since three pairwise comparisons were performed for each variable, the Bonferroni-adjusted threshold for statistical significance was set at *p* < 0.0167. For pairwise comparisons of binary variables, absolute differences in proportions with Newcombe–Wilson 95% confidence intervals were reported as effect estimates. A two-sided *p* value of <0.05 was considered statistically significant for overall comparisons. Given the small sample size, all comparative analyses were considered exploratory.

## 3 Results

### 3.1 Baseline characteristics

During the study period, serum TPHA testing was performed in 9,172 hospitalized neurology patients. Twenty-eight patients had positive serum treponemal serology, corresponding to a seropositivity frequency of 0.31%. Of these, three were excluded because of incomplete clinical data, and the remaining 25 patients met the eligibility criteria and were included in the final analysis.

Of the 25 patients, 10 were classified as confirmed neurosyphilis, 5 as probable neurosyphilis, and 10 as non-neurosyphilis seropositivity. The demographic, clinical, radiological, and discharge characteristics of the study cohort according to diagnostic group are presented in Table 2.

**Table 2:** Demographic, clinical, radiological, and discharge characteristics according to diagnostic group.

Parameter	All patients (*n* = 25)	Non-neurosyphilis seropositivity (*n* = 10)	Probable neurosyphilis (*n* = 5)	Confirmed neurosyphilis (*n* = 10)	*p*-value
Age, years, mean ± SD	52.8 ± 15.3	53.1 ± 16.2	49.2 ± 11.0	54.3 ± 17.3	0.841
Male sex, *n* (%)	22 (88.0)	8 (80.0)	5 (100.0)	9 (90.0)	0.515
Vascular ischemia on admission, *n* (%)	12 (48.0)	4 (40.0)	3 (60.0)	5 (50.0)	0.755
HIV positive, *n* (%)	3 (12.0)	0 (0.0)	1 (20.0)	2 (20.0)	0.321
MRI findings suggestive of neurosyphilitic involvement, *n* (%)	16 (64.0)	2 (20.0)	4 (80.0)	10 (100.0)	0.001
CTA vascular involvement, *n* (%)	7 (28.0)	1 (10.0)	1 (20.0)	5 (50.0)	0.125
*Positive neurological examination at discharge, *n* (%)	15 (62.5)	2 (20.0)	4 (80.0)	9 (100.0)	0.001
Strongly positive serum VDRL (≥1/16), *n* (%)	6 (27.3)	1 (12.5)	0 (0.0)	5 (55.6)	0.041

Values are presented as mean ± standard deviation or number (%). CTA, computed tomography angiography; HIV, human immunodeficiency virus; MRI, magnetic resonance imaging; SD, standard deviation. *Neurological status at discharge was evaluated only among patients discharged alive; one patient who died in hospital was excluded from this analysis. Serum VDRL titer data were unavailable for three patients; and the p value for serum VDRL was obtained using Pearson’s chi-square test and should be interpreted cautiously because of small expected cell counts.

There were no statistically significant differences among the three diagnostic groups in terms of age, sex distribution, vascular ischemia on admission, HIV status, or CTA vascular involvement. HIV positivity was detected in three patients, including one patient in the probable neurosyphilis group and two patients in the confirmed neurosyphilis group. Among the three HIV-positive patients, two had a positive neurological examination at discharge, while one died during hospitalization.

### 3.2 MRI findings

Among the MRI findings considered suggestive of neurosyphilitic involvement, a meningovascular pattern was observed in 13 patients, including two with hemorrhagic infarction. Diffuse cerebral atrophy suggestive of parenchymal involvement was identified in two patients, while spinal MRI abnormalities suggestive of neurosyphilitic involvement were observed in one patient. For both MRI findings suggestive of neurosyphilitic involvement and positive neurological examination at discharge, the overall comparison among the three diagnostic groups was statistically significant (*p* = 0.001; Table 2). *Post hoc* pairwise comparisons were performed using two-sided Fisher’s exact tests with a Bonferroni-adjusted significance threshold of *p* < 0.0167. MRI findings suggestive of neurosyphilitic involvement were more frequent in the confirmed neurosyphilis group than in the non-neurosyphilis seropositivity group (10/10 [100%] vs. 2/10 [20%]; *p* = 0.0007). The differences between probable neurosyphilis and non-neurosyphilis seropositivity (4/5 [80%] vs. 2/10 [20%]; *p* = 0.0889) and between confirmed and probable neurosyphilis (10/10 [100%] vs. 4/5 [80%]; *p* = 0.3333) were not statistically significant after Bonferroni correction. Among the 22 patients with available serum VDRL titers, the distribution of titers <1:16 and ≥1:16 differed across the diagnostic groups in the exploratory Pearson chi-square analysis (χ^2^(2) = 6.385, *p* = 0.041). However, this finding should be interpreted cautiously because 66.7% of the expected cell counts were below 5. In *post hoc* pairwise comparisons using two-sided Fisher’s exact tests with Bonferroni correction, no comparison reached statistical significance: confirmed neurosyphilis versus non-neurosyphilis seropositivity, *p* = 0.131; probable neurosyphilis versus non-neurosyphilis seropositivity, *p* = 1.000; and confirmed versus probable neurosyphilis, *p* = 0.086. One patient in the confirmed neurosyphilis group died.

MRI findings suggestive of neurosyphilitic involvement and positive neurological examination at discharge showed significant overall differences among the diagnostic groups, with significant pairwise differences observed only between the confirmed neurosyphilis and non-neurosyphilis seropositivity groups. Detailed pairwise effect estimates with 95% confidence intervals are presented in Table 3.

**Table 3:** Pairwise effect estimates for binary variables.

Outcome	Comparison	Absolute difference in proportions (95% CI)	Two-sided Fisher’s exact *p*-value
Male sex	Confirmed vs. non-neurosyphilis	0.10 (−0.24 to 0.42)	1.000
	Probable vs. non-neurosyphilis	0.20 (−0.26 to 0.51)	0.524
	Confirmed vs. probable	−0.10 (−0.40 to 0.34)	1.000
Vascular ischemia on admission	Confirmed vs. non-neurosyphilis	0.10 (−0.29 to 0.45)	1.000
	Probable vs. non-neurosyphilis	0.20 (−0.27 to 0.57)	0.608
	Confirmed vs. probable	−0.10 (−0.49 to 0.35)	1.000
HIV positivity	Confirmed vs. non-neurosyphilis	0.20 (−0.11 to 0.51)	0.474
	Probable vs. non-neurosyphilis	0.20 (−0.12 to 0.62)	0.333
	Confirmed vs. probable	0.00 (−0.45 to 0.35)	1.000
MRI findings suggestive of neurosyphilitic involvement	Confirmed vs. non-neurosyphilis	0.80 (0.38 to 0.94)	0.0007
	Probable vs. non-neurosyphilis	0.60 (0.07 to 0.82)	0.089
	Confirmed vs. probable	0.20 (−0.12 to 0.62)	0.333
CTA vascular involvement	Confirmed vs. non-neurosyphilis	0.40 (−0.00 to 0.68)	0.141
	Probable vs. non-neurosyphilis	0.10 (−0.25 to 0.53)	1.000
	Confirmed vs. probable	0.30 (−0.20 to 0.61)	0.580
*Positive neurological examination at discharge	Confirmed vs. non-neurosyphilis	0.80 (0.37 to 0.94)	0.0007
	Probable vs. non-neurosyphilis	0.60 (0.07 to 0.82)	0.089
	Confirmed vs. probable	0.20 (−0.14 to 0.62)	0.357
Serum VDRL ≥1:16	Confirmed vs. non-neurosyphilis	0.43 (−0.02 to 0.71)	0.131
	Probable vs. non-neurosyphilis	−0.12 (−0.47 to 0.32)	1.000
	Confirmed vs. probable	0.56 (0.03 to 0.81)	0.086

Values are the proportion in the first-named group minus the proportion in the second-named group. Confidence intervals were calculated using the Newcombe–Wilson method. *p*-values were obtained using two-sided Fisher’s exact tests. A Bonferroni-adjusted threshold of *p* < 0.0167 was applied to the three pairwise comparisons for each outcome. Estimates should be interpreted cautiously because of the small sample size and sparse cells; all pairwise comparisons should be considered exploratory. Serum VDRL data were available for 22 patients.

*Neurological status at discharge was evaluated only among patients discharged alive; one patient who died in hospital was excluded from this analysis.

### 3.3 Individual patient characteristics

The individual clinical, laboratory, radiological, and outcome characteristics of the 25 patients are summarized in Table 4. Clinical presentations were diverse and included acute ischemic stroke, transient ischemic attack, intracerebral hemorrhage, epileptic seizure, cognitive and behavioral changes, headache, myelopathy, and dizziness. Neuroimaging findings also covered a wide range of abnormalities, from normal examinations and nonspecific white matter changes to meningovascular infarctions, diffuse cerebral atrophy, spinal cord involvement, and hemorrhagic infarction. Cerebrospinal fluid findings and serum nontreponemal titers also varied across patients, reflecting the diversity of neurological manifestations associated with syphilis seropositivity. Neurological examination findings at discharge ranged from the absence of focal neurological deficits to persistent cognitive impairment, hemiparesis, gait ataxia and visual field defects; one patient died during hospitalization.

**Table 4:** Individual clinical, laboratory, radiological, and outcome data of the study cohort (*n* = 25).

Pt No	Age/sex	Clinical presentation	Serum TPHA	Quantitative serum VDRL titer	CSF VDRL	CSF protein (g/L)	CSF cell count (cells/ μL)	HIV infection	MRI findings	CTA findings	Diagnostic group	IV antibiotic treatment (penicillin or ceftriaxone)	Neurological status at discharge	In-hospital death
1	51/M	Acute ischemic stroke	Positive	1:16	Positive	0.72	18	Negative	Right PCA infarct	Right PCA occlusion	Confirmed	Penicillin	Left homonymous hemianopsia	No
2	70/M	Cognitive and behavioral changes	Positive	1:16	Positive	0.68	14	Negative	Diffuse atrophy	Normal	Confirmed	Penicillin	Cognitive impairment with disorientation	No
3	52/M	Acute ischemic stroke	Positive	1:1	Negative	0.52	6	Negative	Left pons infarct	Normal	Probable	None	Dysarthria and gait ataxia	No
4	61/M	Acute ischemic stroke	Positive	1:1	Negative	0.51	7	Negative	Right thalamic infarct	Right PCA (P1) focal narrowing	Probable	Ceftriaxone	Left hemiparesis	No
5	54/M	Acute ischemic stroke	Positive	1:64	Negative	0.33	0	Negative	Left frontal lobe infarct	Left M2 branch occlusion	Non-NS	None	Mild right hemiparesis	No
6	60/M	Epileptic seizure	Positive	1:16	Positive	0.48	2	Negative	Left pons infarct	Normal	Confirmed	Penicillin	Dysarthria and gait ataxia	No
7	54/M	Epileptic seizure	Positive	1:4	Negative	0.30	0	Negative	Non-specific white matter changes	Normal	Non-NS	None	No focal neurological deficit	No
8	68/M	Transient ischemic attack	Positive	1:4	Negative	0.35	1	Negative	Non-specific white matter changes	Normal	Non-NS	None	No focal neurological deficit	No
9	51/M	Acute ischemic stroke	Positive	N/A	Positive	0.83	26	Negative	Left MCA infarct	Left MCA (M1) stenosis	Confirmed	Ceftriaxone	Right hemiparesis	No
10	26/M	Acute ischemic stroke	Positive	1:32	Positive	1.44	61	Positive	Left MCA infarct	Left MCA occlusion	Confirmed	Penicillin	N/A	Yes
11	28/M	Headache	Positive	1:1	Negative	0.24	1	Negative	Non-specific white matter changes	Normal	Non-NS	None	No focal neurological deficit	No
12	54/M	Intracerebral hemorrhage	Positive	1:1	Negative	0.46	6	Negative	Right thalamic hematoma	Normal	Non-NS	None	No focal neurological deficit	No
13	36/F	Myelopathy	Positive	N/A	Negative	0.35	1	Negative	Normal	Normal	Non-NS	None	Left lower extremity weakness	No
14	22/M	Myelopathy	Positive	1:16	Positive	0.65	10	Positive	Cervical dorsal T2 hyperintensity	Normal	Confirmed	Ceftriaxone	Sensory ataxia, Romberg (+)	No
15	50/M	Headache	Positive	1:1	Negative	0.33	0	Negative	Non-specific white matter changes	Normal	Non-NS	None	No focal neurological deficit	No
16	55/M	Transient ischemic attack	Positive	N/A	Negative	0.37	1	Negative	Non-specific white matter changes	Normal	Non-NS	None	No focal neurological deficit	No
17	31/M	Cognitive and behavioral changes	Positive	1:1	Negative	0.62	8	Positive	Fronto-temporal atrophy	Normal	Probable	Penicillin	Cognitive impairment	No
18	51/M	Dizziness	Positive	1:1	Negative	0.52	7	Negative	Non-specific white matter changes	Normal	Probable	None	Mild gait ataxia	No
19	45/M	Headache	Positive	1:1	Negative	0.54	5	Negative	Non-specific white matter changes	Normal	Non-NS	None	No focal neurological deficit	No
20	66/M	Acute ischemic stroke	Positive	1:4	Positive	0.57	8	Negative	Right temporal punctate infarct	Normal	Confirmed	Penicillin	Left hemisensory deficit	No
21	64/M	Acute ischemic stroke	Positive	1:1	Positive	0.82	22	Negative	Right MCA infarct	Right MCA M1- M2 segmental stenosis	Confirmed	Penicillin	Left hemiparesis	No
22	87/F	Transient ischemic attack	Positive	1:1	Negative	0.24	1	Negative	Non-specific white matter changes	Normal	Non-NS	None	No focal neurological deficit	No
23	63/F	Cognitive and behavioral changes	Positive	1:8	Positive	0.73	11	Negative	Left MCA infarct	Left MCA M1-M2 occlusion	Confirmed	Penicillin	Right hemiplegia and global aphasia	No
24	51/M	Acute ischemic stroke	Positive	1:2	Negative	0.49	6	Negative	Right centrum semiovale infarct	Normal	Probable	Penicillin	No focal neurological deficit	No
25	70/M	Hemorrhagic infarction	Positive	1:1	Positive	0.63	9	Negative	Left globus pallidus hemorrhagic infarct	Normal	Confirmed	Penicillin	Right hemiparesis	No

Pt No: patient number; M: male; F: female; VDRL: venereal disease research laboratory; CSF: cerebrospinal fluid; MRI: magnetic resonance imaging; CTA: computed tomography angiography; PCA: posterior cerebral artery; MCA: middle cerebral artery; HIV: human immunodeficiency virus; IV: intravenous; N/A: not available; Non-NS: non-neurosyphilis seropositivity note: P1, M1, and M2 refer to specific anatomical segments of the posterior and middle cerebral arteries identified during neuroimaging.

### 3.4 Treatment and neurological outcomes

Among the 10 patients with confirmed neurosyphilis, eight received the standard regimen of intravenous aqueous crystalline penicillin G (24 million units/day for 14 days). The remaining two patients had documented penicillin allergy and were treated with intravenous ceftriaxone (2 g once daily, administered intravenously, for 14 days), in accordance with contemporary guideline recommendations.

Five patients were classified as having probable neurosyphilis. Two received the standard regimen of intravenous aqueous crystalline penicillin G, while one patient with documented penicillin allergy was treated with intravenous ceftriaxone. The remaining two patients were referred to the Infectious Diseases outpatient clinic after hospital discharge for further evaluation and stage-appropriate syphilis treatment based on the overall clinical, serological and cerebrospinal fluid findings.

None of the patients classified as having non-neurosyphilis seropositivity received neurosyphilis-directed intravenous antimicrobial therapy during hospitalization, as they lacked sufficient clinical and cerebrospinal fluid evidence of central nervous system involvement. Instead, all patients in this group were referred to the Infectious Diseases outpatient clinic after discharge for comprehensive evaluation and stage-appropriate syphilis treatment.

Neurological status at hospital discharge varied considerably across the study cohort. Eight patients were discharged without residual focal neurological deficits. Among the remaining patients, persistent neurological impairment most commonly manifested as hemiparesis in five patients, cognitive impairment with or without disorientation in three patients, gait ataxia in three patients, and dysarthria in two patients. Less frequent residual deficits included left homonymous hemianopia, left hemisensory deficit, right hemiplegia accompanied by global aphasia, left lower extremity weakness, and sensory ataxia with a positive Romberg sign, each observed in one patient. One patient with confirmed neurosyphilis died during hospitalization despite receiving appropriate antimicrobial therapy. Overall, neurological outcomes ranged from complete clinical recovery without detectable neurological deficits to persistent motor, sensory, visual, cognitive, and cerebellar impairments, with one in-hospital death. Detailed discharge neurological findings for each patient are presented in Table 4.

## 4 Discussion

In this single-center retrospective study, syphilis seropositivity was identified in 28 of 9,172 patients hospitalized in the neurology ward, corresponding to a frequency of 0.31%. After exclusion of three patients with incomplete clinical data, 25 eligible patients were included in the final analysis. Among these seropositive patients, 60% were classified as having confirmed or probable neurosyphilis. The main findings were that MRI findings suggestive of neurosyphilitic involvement and positive neurological examination at discharge differed significantly among the diagnostic groups, whereas age, sex, vascular ischemia on admission, HIV status, and CTA vascular involvement did not. Pairwise analyses showed that these differences were driven by the contrast between confirmed neurosyphilis and non-neurosyphilis seropositivity.

The low frequency of syphilis seropositivity in our hospitalized neurology cohort is consistent with the fact that neurosyphilis remains an uncommon diagnosis in routine neurological practice. However, the proportion of seropositive patients who fulfilled confirmed or probable neurosyphilis criteria was clinically meaningful. This finding supports the continued relevance of syphilis testing in selected neurological patients, particularly because syphilis has re-emerged globally and may involve the nervous system at any stage of infection (1, 2, 5, 6). The neurological manifestations of syphilis are broad and may include asymptomatic CSF abnormalities, meningitis, meningovascular disease, ocular or otic involvement, psychiatric symptoms, cognitive impairment, myelopathy, and parenchymal forms such as general paresis and tabes dorsalis (1, 5, 6, 11–13).

A key issue highlighted by this study is the practical importance of diagnostic stratification. Neurosyphilis diagnosis remains difficult because there is no universally accepted gold standard, and the available diagnostic tools have variable performance (7, 8). Boog et al. emphasized that the literature uses heterogeneous reference standards and that the current basis for diagnosis remains clinical suspicion supported by CSF analysis (8). Similarly, Zhang et al. noted that clinical practice is complicated by the lack of highly efficient diagnostic criteria and by the limitations of CSF-based testing (7). In this context, categorizing patients as confirmed, probable, presumptive, likely, or non-neurosyphilis seropositive is not merely semantic; it reflects the diagnostic uncertainty that clinicians face in real-world practice (6, 7, 9–11).

The distinction between confirmed and probable neurosyphilis is especially relevant because a negative CSF-VDRL does not exclude neurosyphilis. Although CSF-VDRL is highly specific and strongly supports the diagnosis when positive, its sensitivity is limited (2, 6–9). Dyckhoff-Shen et al. reported that CSF-VDRL was negative in nearly half of patients with confirmed neurosyphilis, reinforcing the need for multimodal assessment (6). Xiao et al. also used a diagnostic framework that separated confirmed and probable neurosyphilis among HIV-negative syphilis patients with neurological symptoms and compared these patients with a syphilis/non-neurosyphilis group (9). The definitions of confirmed and probable neurosyphilis used in this study were based on established diagnostic criteria, with the probable neurosyphilis category largely following the CDC surveillance definition. Under the CDC surveillance definition, probable neurosyphilis can be considered in seropositive patients with compatible neurological manifestations and a nonreactive CSF-VDRL when CSF pleocytosis (>5 cells/μL) and/or elevated CSF protein (>0.50 g/L) is present without another known cause (14). The German Society for Neurology guideline also emphasizes the combined evaluation of clinical presentation, serum treponemal serology, and CSF findings, although its approach to probable neurosyphilis differs from the CDC definition and also takes clinical course and response to treatment into account (15). Thus, while both recommendations support the interpretation of neurological, serological, and CSF findings together, the specific criteria are not identical. The non-neurosyphilis seropositivity group was not derived from either guideline but was used as a study-specific descriptive category for seropositive patients who did not meet the predefined criteria for confirmed or probable neurosyphilis, allowing comparison within this retrospective cohort without implying a new diagnostic classification.

MRI findings suggestive of neurosyphilitic involvement were significantly more frequent in the confirmed neurosyphilis group than in the non-neurosyphilis seropositivity group. Neuroimaging findings in neurosyphilis are not pathognomonic and may vary according to the clinical subtype, but they can provide important supportive evidence when interpreted together with clinical and CSF findings (6, 8, 12, 16). Previous studies have reported heterogeneous neuroimaging findings in neurosyphilis, ranging from ischemic lesions and meningeal or contrast-enhancing abnormalities to atypical MRI patterns that may mimic inflammatory, vascular, or degenerative neurological disorders (6, 16–18). In our cohort, the significant difference in MRI involvement suggests that radiological assessment may help distinguish clinically relevant CNS disease among seropositive neurology inpatients, although MRI should not be considered diagnostic in isolation.

Positive neurological examination at discharge was also significantly more frequent in confirmed neurosyphilis. This finding indicates that patients with confirmed neurosyphilis may have a greater short-term neurological burden than seropositive patients without clinical and CSF evidence of neurosyphilis. Prior studies have shown that symptomatic neurosyphilis may be associated with persistent neurological sequelae, particularly in patients with parenchymal disease, meningovascular involvement, elevated CSF protein, or marked CSF inflammatory abnormalities (11, 12, 19). Li et al. reported that symptomatic neurosyphilis was associated with higher CSF white blood cell count, higher CSF protein concentration, and higher CSF RPR positivity compared with asymptomatic neurosyphilis (11). In another large cohort, symptomatic neurosyphilis was associated with male sex, lack of prior antisyphilis treatment, high pretreatment serum RPR titer, and positive CSF RPR (12). In our study, the worse discharge neurological status in confirmed cases is consistent with the broader literature showing that symptomatic and diagnostically definite neurosyphilis may carry substantial morbidity.

Vascular ischemia on admission and CTA vascular involvement did not differ significantly among groups. This finding should be interpreted cautiously because the small sample size limited statistical power and because vascular presentations are common in neurology inpatient settings for reasons unrelated to neurosyphilis. Nevertheless, meningovascular neurosyphilis remains an important consideration in selected patients with stroke. Ahbeddou et al. described 53 patients with syphilitic arteritis and ischemic stroke and recommended that neurosyphilis testing be considered in neurology departments, particularly to avoid delayed diagnosis (17). Recent reviews have also emphasized that meningovascular neurosyphilis may mimic common cerebrovascular disorders and that diagnosis requires integration of serological, CSF, neuroimaging, and vascular imaging findings (18, 20). In our cohort, the lack of a statistically significant difference in vascular ischemia does not exclude a role for meningovascular disease, but it suggests that vascular presentation alone may not be sufficient to distinguish neurosyphilis from non-neurosyphilis seropositivity in a hospitalized neurology population.

HIV positivity was observed in three patients, all of whom were in the probable or confirmed neurosyphilis groups, but HIV status did not differ significantly among diagnostic categories. Of the three HIV-positive patients, two had positive neurological examination findings at discharge, while one died during hospitalization. This observation should not be overinterpreted because of the very small number of HIV-positive patients. Larger studies have reported variable relationships between HIV infection and neurosyphilis presentation or outcome. Dyckhoff-Shen et al. found a high proportion of HIV coinfection in their neurosyphilis cohort and reported more pronounced CSF abnormalities and additional coinfections among people living with HIV (6). In contrast, Schnohr et al. reported that people living with HIV had fewer neurological deficits and no unfavorable outcomes at discharge in a Danish nationwide cohort (19). These differences likely reflect variation in case mix, immune status, timing of diagnosis, treatment access, and cohort setting. Therefore, our data are insufficient to draw conclusions regarding the prognostic effect of HIV infection.

The probable neurosyphilis group deserves particular attention. In our study, this group often showed an intermediate pattern between confirmed neurosyphilis and non-neurosyphilis seropositivity, but pairwise comparisons did not show statistically significant differences after Bonferroni correction. This may reflect limited statistical power rather than absence of clinical relevance. Studies using probable, presumptive, or likely categories have emphasized that patients without a positive CSF-VDRL may still have clinically meaningful CNS involvement when neurological manifestations and inflammatory CSF abnormalities coexist (6, 9–11, 15, 21). Thus, probable neurosyphilis should be viewed as a clinically important diagnostic category, particularly in retrospective real-world cohorts where complete CSF and advanced laboratory data may not always be available.

This diagnostic classification also has potential therapeutic implications. Current guidelines recommend aqueous crystalline penicillin G, 18–24 million units per day intravenously for 10–14 days, for neurosyphilis (2). However, because CSF-VDRL has limited sensitivity and no single laboratory test can reliably establish or exclude neurosyphilis in every clinical setting, treatment decisions should integrate neurological manifestations, serum serology, and inflammatory CSF findings rather than rely on CSF-VDRL alone (5, 22). In patients with strong clinical suspicion and inconclusive CSF findings, delayed treatment may risk irreversible neurological injury, whereas the benefit of neurosyphilis-directed treatment in asymptomatic seropositive patients with isolated CSF abnormalities remains uncertain (22–24).

This study has several limitations. First, it was retrospective and single-center, which may limit generalizability. Second, the final sample size was small, especially in the probable neurosyphilis group, and all comparative analyses should therefore be interpreted as exploratory. Third, the final analysis included only selected clinical and radiological variables; treatment response, and long-term outcomes were not included. Fourth, MRI and CTA findings were analyzed as binary variables, which may not fully capture lesion distribution, vascular patterns, or radiological severity. Because of the retrospective design, the exact timing of MRI acquisition relative to symptom onset could not be determined consistently from the medical records. Nevertheless, MRI examinations were obtained as part of the routine diagnostic evaluation during hospitalization In addition, given the clinical relevance of sexually transmitted coinfections in patients with syphilis, their assessment would have been valuable. However, because this study was conducted retrospectively in a neurology inpatient setting, routine screening for sexually transmitted infections other than HIV was not systematically performed, and these data were therefore not consistently available. Finally, because the study included only hospitalized neurology patients, the results may not apply to outpatient, dermatology, infectious disease, ophthalmology, or psychiatry-based populations.

In conclusion, this 16-year retrospective study demonstrates that although syphilis seropositivity was uncommon among hospitalized neurology patients (0.31%), 60% of analyzed seropositive patients fulfilled predefined diagnostic criteria for confirmed or probable neurosyphilis. These findings underscore that syphilis seropositivity should not be considered synonymous with neurosyphilis and highlight the importance of a comprehensive diagnostic evaluation integrating clinical presentation, cerebrospinal fluid findings, and neuroimaging. For descriptive purposes, patients in this retrospective cohort were categorized into confirmed neurosyphilis, probable neurosyphilis, and non-neurosyphilis seropositivity. Within this cohort, MRI findings suggestive of neurosyphilitic involvement and positive neurological examination findings at discharge were more frequently observed among patients with confirmed neurosyphilis. Furthermore, syphilis testing remains a crucial component in the diagnostic evaluation of unexplained neurological syndromes in contemporary practice. Given the retrospective design and limited sample size, this descriptive categorization should be interpreted cautiously larger multicenter studies incorporating detailed CSF parameters, treatment data, and long-term outcomes are needed to further refine diagnostic strategies, validate the reproducibility of this descriptive categorization, and better define the clinical significance of probable neurosyphilis.

## Data Availability

The raw data supporting the conclusions of this article will be made available by the authors, without undue reservation.
